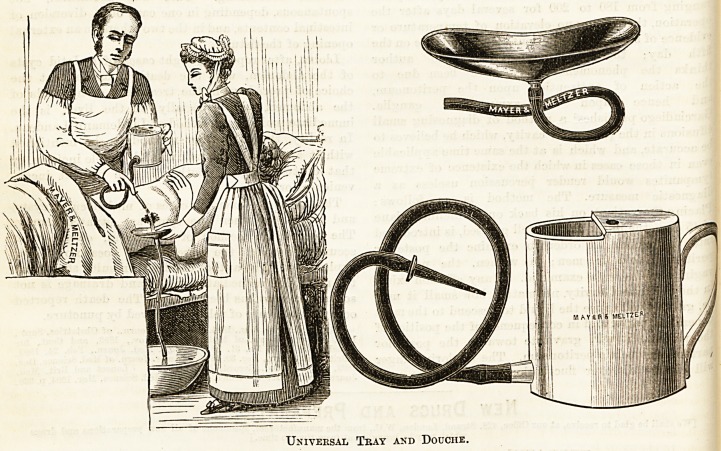# New Appliances and Things Medical

**Published:** 1894-04-21

**Authors:** 


### NEW APPLIANCES AND THINGS MEDICAL.

[All preparations, appliances, novelties, &c., of which a notice is desired, should be sent for the Editor, to care of The Manager, 428, Strand, London, W.C.]

### THE UNIVERSAL DRESSING TRAY.

(Mayer and Meltzer, 71, Great Portland Street, W.)

A reference to the diagram will explain the use of this contrivance. Perhaps the most remarkable feature of the invention is that it has never been thought of before. The old pus tray was a clumsy concern, and although everybody knew this, everybody continued to use it. The great value of this tray is that from start to finish it need never be moved from the position in which it is first applied, and hence the contents cannot be upset over the bedding or clothes of the patient; and since the tray empties itself as fast as the fluids drain into it, under ordinary circumstances it can never overflow. There can be no question that this will be the dressing tray of the future. At present the tray is made of polished metal; it occurs to us that for some purposes a china recipient might be a more cleanly utensil.

Universal Tray and Douche.

### MINERAL WATERS, &c.

(R. Ellis and Son, Ruthin.)

The above firm have forwarded us samples of their various mineral waters. Their list, which is a goodly one, includes the following: 1, Soda water; 2, potass water; 3, Lithia water; 4, seltzer water; 5, ginger ale, non-alcoholic; 6, ginger ale, aromatic; 7, brewed ginger ale. We have examined these waters, and can detect the presence of no objectionable preservatives nor noxious chemical substances, such as are occasionally added by manufacturers in the preparation of these beverages. Those who like to observe the outward form of temperance disciples, and yet get just a spice of alcohol, will do well to drink the brewed ginger beer. They will thus be able to enjoy the spirit while observing the letter of the law. The ginger ale, on the other hand, is non-alcoholic, and is an excellent temperance beverage. With regard to their mineral waters—Lithia, soda, and potass—they all appear excellent of their kind.

## Figures and Tables

**Figure f1:**